# Lowly Expressed Ribosomal Protein S19 in the Feces of Patients with Colorectal Cancer

**DOI:** 10.5402/2012/394545

**Published:** 2012-01-09

**Authors:** Chih-Cheng Chien, Tien-Chien Tu, Chi-Jung Huang, Shung-Haur Yang, Chia-Long Lee

**Affiliations:** ^1^Department of Anesthesiology, Sijhih Cathay General Hospital, New Taipei 22174, Taiwan; ^2^School of Medicine, Fu Jen Catholic University, New Taipei 24257, Taiwan; ^3^Department of Internal Medicine, Cathay General Hospital, Taipei 10630, Taiwan; ^4^School of Medicine, Taipei Medical University, Taipei 11031, Taiwan; ^5^Department of Medical Research, Cathay General Hospital, Taipei 10630, Taiwan; ^6^Department of Biochemistry, National Defense Medical Center, Taipei 11490, Taiwan; ^7^Department of Surgery, Taipei-Veterans General Hospital, Taipei 11217, Taiwan; ^8^School of Medicine, National Yang Ming University, Taipei 11221, Taiwan; ^9^Department of Internal Medicine, Hsinchu Cathay General Hospital, Hsinchu 30060, Taiwan

## Abstract

Colorectal cancer (CRC) has become one of the most common fatal cancers. CRC tumorigenesis is a complex process involving multiple genetic changes to several sequential mutations or molecular alterations. P53 is one of the most significant genes; its mutations account for more than half of all CRC. Therefore, understanding the cellular genes that are directly or indirectly related to p53 is particularly crucial for investigating CRC tumorigenesis. In this study, a p53-related ribosomal protein, ribosomal protein S19 (RPS19), obtained from the feces of CRC patients is evaluated by using specifically quantitative real-time PCR and knocked down in the colonic cell line by gene silencing. This study found that CRC patients with higher expressions of RPS19 in their feces had a better prognosis and consistent expressions of RPS19 and BAX in their colonic cells. In conclusion, the potential mechanism of RPS19 in CRC possibly involves cellular apoptosis through the BAX/p53 pathway, and the levels of fecal RPS19 may function as a prognostic predictor for CRC patients.

## 1. Introduction

Despite progress in reducing the incidence and mortality rate and improving patient survival, human cancers still account for numerous deaths [1]. Colorectal cancer (CRC) has become one of the most common fatal cancers, involving a complex process with multiple genetic changes [2–4]. This molecular heterogeneity possibly results from multiple sequential mutations or molecular alterations during tumorigenesis [5]. Therefore, the identification of CRC-related genes will assist in cancer prevention, detection, and prognostic prediction [6–8].

One important tumor suppressor, p53, is known to prevent cancer, but is also involved in CRC progression [9, 10]. Mutations of p53 account for more than half of all CRCs, particularly in patients at the more advanced stages [11]. Numerous cellular genes are also out of control because of the abnormal p53 expression during tumor progression [12, 13]. For example, the p53-related ribosomal proteins (RPs) were identified as cancer-related molecules [14, 15], indicating that the oncogenic potential of RPs result from the relationship between p53 and RPs [16–18]. Moreover, the p53-inducible modulator RPS27-like (RPS27L), which responds to genotoxic stress, was recently evaluated in CRC [19].

Feces can serve as the material for detecting genetic alterations in CRC [20–22]. Numerous ribosomal proteins are significantly expressed in the feces of CRC patients [23]. In this study, p53-related RPS19 of CRC was further evaluated. Other studies have reported on the developmental abnormalities resulting from a RPS19 deficiency through the activation of the p53 protein family [24]. First, the clinical significance of RPS19 in feces was evaluated from the stool samples of CRC patients using specifically, quantitative real-time PCR (qRT-PCR). Then, the functional importance of RPS19 was addressed by silencing its expression in colonic cells. In this context, we explored the possible cell fate of changing the RPS19 expression in colonic cells, which could affect CRC patients' survival.

## 2. Materials and Methods

### 2.1. Patients

Solid fecal samples of 101 CRC patients (*n*_male_ = 69; *n*_female_ = 32) from the Cathay General Hospital and the Taipei Veterans General Hospital were obtained before surgery or application of chemotherapy, with IRB-approved informed consent. Follow-up data were obtained prospectively, and the mean follow-up time was 44.1 months (SD, 29.0; median, 37.8). Patients' initial tumor stage and additional clinical information are listed in Table 1. Patients with distant metastasis were routinely confirmed by abdominal computed tomography.

### 2.2. Total RNA Extraction and Reverse Transcription Reaction

The HCT116 cell line was cultured in Dulbecco's modified Eagle's medium with 5 mM of glutamine according to routine culture procedures. RNA from this cell line was extracted using the Easy Pure Total RNA Mini Kit (Bioman, Taiwan) and reverse transcribed for single-stranded cDNAs using an oligo(dT)_12_ primer with the ABI Reverse Transcriptase kit (ABI, USA), according to the manufacturer's protocols [25]. Fecal RNA was prepared and reverse transcribed as described in our previous reports [22]. In brief, synthesized cDNA could be used directly in the following quantitative PCR analyses.

### 2.3. Quantitative Real-Time PCR (qRT-PCR)

The quantitations of RPS19 (NM001022), BAX (NM138764), and 18s rRNA (X03205) in fecal cDNA were performed using a TaqMan probe (probe no. 87 for RPS19, no. 55 for BAX, and no. 77 for 18 s rRNA) from the Human Universal Probe Library (Roche Diagnostics, Germany). The 18 s rRNA served as a housekeeping gene. Generally, each run of fecal samples must include the human reference cDNA (Clontech, USA) as standard to avoid errors because of run-to-run differences in RNA quantity. The primer sequences for these quantitations are listed in Table 2.

### 2.4. Lentivirus-Mediated RNA Interference (RNAi) of RPS19

The lentiviral construct encoding the siRPS19 hairpin (pLKO.1-RPS19 : TRCN0000074915) for gene silencing (shRPS19) was obtained from the National RNAi Core Facility located at the Institute of Molecular Biology/Genomic Research Center, Academia Sinica, Taipei Taiwan. Additionally, the control (shLuc) for the lentivirus was pLKO.1-Luc (TRCN0000072246), and the infection of each lentivirus into colonic cells was performed according to our previous report [20]. The change in the expression of target RPS19 was quantified by qRT-PCR as previously described and immunodetected using western blotting as the routine procedure with minor modifications [25]. Briefly, 5 *μ*g of protein was mixed with the reducing agent NuPAGE SDS sample buffer (Life Technologies, Carlsbad, CA, USA), denatured for 10 min at 95°C, separated by a 12% SDS-PAGE, blotted onto a polyvinylidene difluoride membrane (Millipore, Billerica, MA, USA), and probed with mouse anti-human RPS19 (1 : 1000; sc-100836; Santa Cruz Biotechnology, Santa Cruz, CA, USA) or rabbit anti-human actin (1 : 500; sc-1616-R; Santa Cruz Biotechnology) following standard procedures. The blots were then incubated with anti-mouse (for RPS19) or anti-rabbit (for actin) secondary antibodies (0.2 *μ*g/mL) conjugated to horseradish peroxidase. All western blots were developed using the Western Blot Chemiluminescence Reagent (PerkinElmer Life and Analytical Sciences, Waltham, MA, USA) according to the manufacturer's instructions.

### 2.5. Statistical Analysis

The overall survival probabilities were estimated using the Kaplan-Meier method and compared with the log-rank test using SPSS 13.0 software (SPSS). The MedCalc software statistical package was used to generate a receiver operating characteristic (ROC) curve. Significance was set at *P* < 0.05.

## 3. Results

**Figure 1:**
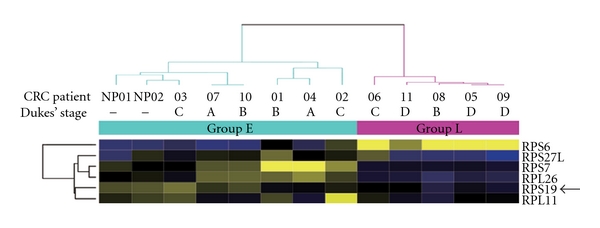
Six differentially expressed p53-associated ribosomal proteins in feces of CRC patients. Differentially expressed genes with statistic significance (*P* < 0.05) are grouped by average-linkage hierarchical clustering. Each row represents a gene and each column is a sample. Group L, five patients (one at Dukes' stage B, one at stage C, and three at stage D). Group E, six patients (two at Dukes' stage A, two at stage B, and two at stage C) and two normal control pools (NP01 and NP02). NP01, pooled by two healthy men; NP02, pooled by three healthy women. A region cluster depicts the genes based on the similarity between their expressions in cases. High expression is shown in deep yellow, low expression in blue. Arrow indicates RPS19.

Over 40 ribosomal or ribosomal-associated genes were clustered due to their significantly differential expressions (*P* < 0.05) in the feces of CRC patients from our previous report [23]. Notably, six p53-associated RPs, including two large- and four small-ribosomal protein transcripts, were clustered by the average-linkage hierarchical clustering method (Figure 1). The 11 CRC patients and two distinct normal pools could be classified into two groups (E and L). First, 80% (4 of 5) of patients of the L group were in the late Dukes' stages (one in Dukes' stage C and three in stage D), and, with the exception of RPS6 (NM001010), their genes were downregulated. Conversely, RPS27L (NM015920), RPS7 (NM001011), and RPL26 (NM000987) were clustered together due to their increasing expressions in the feces of those in group E, which comprised mostly early-stage patients (67%, 4 of 6) and normal controls.

**Figure 2:**
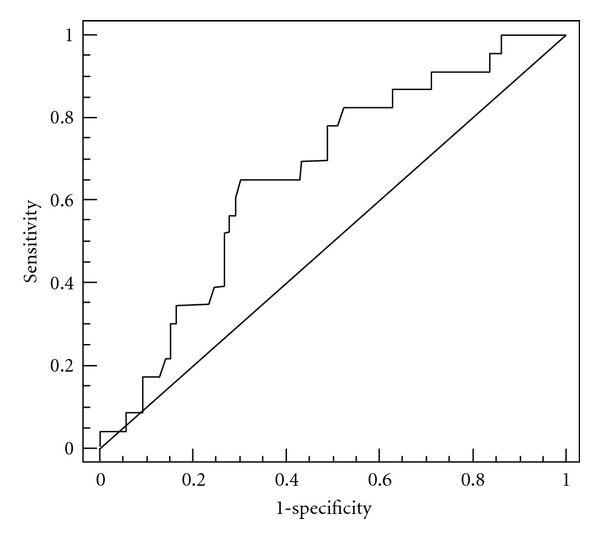
Receiver operating characteristic (ROC) curve for fecal RPS19. The points on the curve represent the relative mRNA levels of RPS19 in the feces and the sensitivity and (1-specificity) of the marker for overall survival.

**Figure 3:**
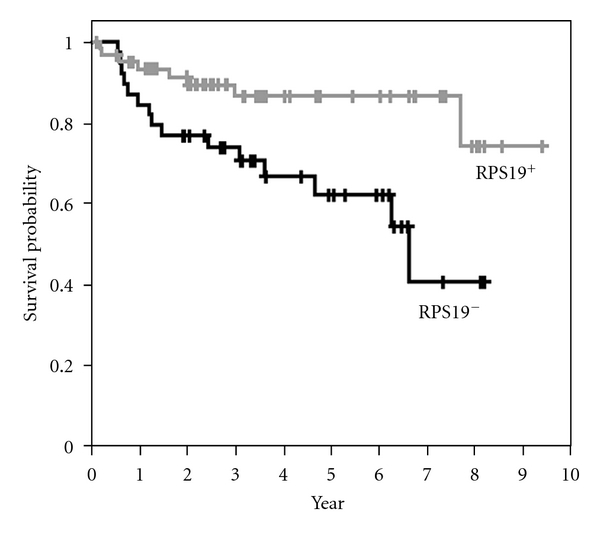
The Kaplan-Meier overall survival curves in patients with colorectal cancer according to fecal RPS19. The relative mRNA levels of RPS19 in the feces are stratified into two groups: RPS19^−^ (<2.76 × 10^−5^) and RPS19^+^ (≥2.76 × 10^−5^). The six-year overall survival rate of the RPS19^+^ group (*n* = 62) is better than that of the RPS19^−^ group (*P* = 0.008, log-rank test).

The clinical significances of RPS19 were further analyzed. The mRNA levels of RPS19 in the feces of CRC patients were stratified into two groups, RPS19^+^ (≥2.76  × 10^−5^) and RPS19^−^ (<2.76 × 10^−5^), using ROC curve analysis. The area under the ROC curve for fecal RPS27L was 0.657 (*P* = 0.012) with a 95% CI of 0.556 to 0.749 (Figure 2). As shown in Figure 3, the RPS19^+^ group (*n* = 62) had the better six-year overall survival rate (74.3 ± 12.2%) than the RPS19^−^ group did (40.9 ± 14.2%; *n* = 39) (*P* = 0.008, log-rank test).

**Figure 4:**
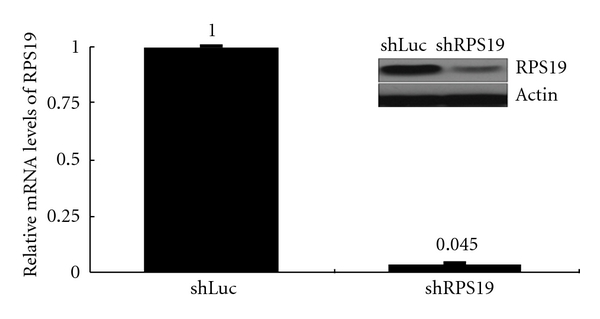
Efficiency of RPS19 silence in colonic cells by RNA interference. RPS19 silence is achieved by the lentivirus-mediated RNAi experiment. Relative mRNA levels of RPS19 are quantified by qRT-PCR with TaqMan probes and normalized by individual level of 18 s rRNA. The relative expression level of shLuc-infected cells is considered as 1. Results are representative of those obtained in two-to-three separate experiments with error bars showing standard error. Changes of protein levels are immunoblotted with antibodies against RPS19 and *β*-actin (in black square).

**Figure 5:**
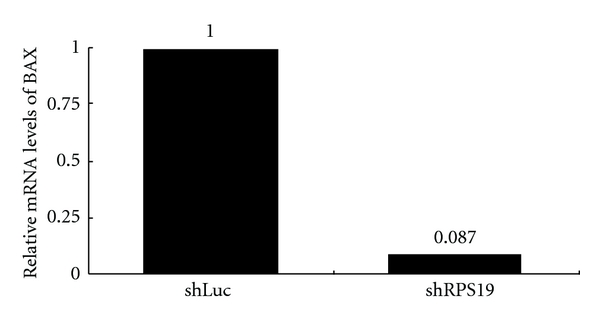
Changes of BAX expression in RPS19-silent colonic cells. Relative mRNA levels of BAX are quantified by qRT-PCR with TaqMan probes and normalized by individual level of 18 s rRNA. The relative expression level of shLuc-infected cells is considered as 1. Results are representative of those obtained in one experiment.

To correlate the cellular function of RPS19, we infected lentiviruses into a colonic cell line with wild-type p53 (HCT116 p53^+/+^) to knockdown RPS19 (shRPS19) and control shRNA (shLuc). In the lentivirus-mediated RNAi experiment, shRPS19 achieved efficient knockdown at both the levels of mRNA and RPS19 protein (Figure 4). These RPS19-silent cells expressed only 9.2% BAX mRNA from RPS19-expressing cells compared to that from RPS19-expressing cells (Figure 5).

## 4. Discussion

Our previous results revealed that numerous fecal molecules were differentially expressed in the feces of CRC patients [26]. Among the fecal molecules, a number of RP genes were listed with statistic significance [23]. RPs are involved not only in the cellular process of translation [27] but also in the growth and maintenance of all cell types [28]. Additionally, numerous reports emphasized that a strong correlation was found between RPs and p53 protein in cellular functions [17, 18, 29]. For example, RPL26 can regulate the translation and induction of p53 after DNA damage [30]. RPS6 participated in the activation of a p53-dependent cell cycle checkpoint [31]. RPS27L was proven to be a p53-inducible modulator of cell fate in response to genotoxic stress [32], and mutant p53 seemed to cause aberrant RPS27L expression, which can lead to the accumulation of tumorigenic CRC cells and a poor prognosis [19].

Besides the p53-associated RPs, other RPs were also revealed to contribute to the onset of cancer or multidrug resistance [33–35]. For example, RPS3 is involved in the onset of cancer [33], RPS13 and RPL23 promote the multidrug resistance of gastric cancer cells [35], and RPL19 is involved in the prognosis of prostate cancer and CRC [23, 36]. Recently, the direct relationship between feces and colonic cells was proven [37, 38]. When combined, detecting fecal RPs, specifically p53-related RPs, might be valuable for evaluating the molecular pathogenesis of CRC [14, 15, 39].

In this study, the overall survival data revealed that CRC patients with higher fecal expressions of RPS19 had a better prognosis. When RPS19 was knocked down in colonic cells, an apoptotic gene (BAX) extremely reduced the expressions in RPS19-silent cells. Nevertheless, most studies of RPS19 examined mutations in the RPS19 gene in patients with Diamond-Blackfan anemia [40, 41]. Cells with an RPS19 deficiency correlate with p53 dysregulation, which may cause developmental anomalies [24]. To our knowledge, we are the first to reveal the differentially expressed RPS19 in CRC with clinical significance. From the results of consistent expressions of RPS19 and BAX, we suggest that downregulated RPS19 might impair the apoptotic function of colonic cells. This argument supports the clinical data that CRC patients with lower fecal expressions of RPS19 had a poor prognosis.

## 5. Conclusions

We succeeded in quantifying the mRNA level of RPS19 in feces. The potential mechanism of RPS19 in CRC possibly involves cellular apoptosis through the BAX/p53 pathway [42]. Our results provide some evidence that the levels of fecal RPS19 may function as a prognostic predictor for CRC patients. Finally, clinical use of feces in translational research is promising for the future of CRC diagnosis [43].

## Figures and Tables

**Table 1:** Clinical characteristics of CRC patients.

Variable	No. of cases	Level of fecal RPS19 (%) (≥2.76 × 10^−5^)	*P*
Age (yr)*			0.448
<64.7	47	27 (57.4)	
≥64.7	54	35 (64.8)	
Gender			0.140
Male	69	39 (56.5)	
Female	32	23 (71.9)	
Depth of invasion			0.303
T1 + T2	15	11 (73.3)	
T3 + T4	86	51 (59.3)	
Lymphatic invasion			0.068
N0	53	37 (69.8)	
N1 + N2 + N3	48	25 (52.1)	
Distant metastasis			0.183
No	84	54 (64.3)	
Yes	17	8 (47.1)	
Tumor size (cm)**			0.682
<4.7	61	36 (59.0)	
≥4.7	38	24 (63.2)	
CEA (ng/mL)**			0.907
≤5	61	37 (60.7)	
>5	37	22 (59.5)	
CA19-9 (U/mL)**			0.829
<37	74	45 (60.8)	
≥37	24	14 (58.3)	

*mean age of 101 patients, 64.7 ys (range, 37.3–89.5). **available cases in tumor size, 99; in serum CEA and CA19-9 determinant, 98.

**Table 2:** Primers' sequences and universal probe numbers for qRT-PCR analysis.

Gene name	Primer sequence*	Probe no.
RPS19	F: 5′-TCAGGGACAAAGAGATCTGGA-3′	87
	R: 5′-CATGGTTTGTTCTAATGCTTCTTG-3′	
BAX	F: 5′-CAAGACCAGGGTGGTTGG-3′	55
	R: 5′-CACTCCCGCCACAAAGAT-3′	
18 s RNA	F: 5′-CTCAACACGGGAAACCTCAC-3′	77
	R: 5′-CGCTCCACCAACTAAGAACG-3′	

*F, forward; R, reverse; Probe no., from the “Human Universal Probe Library” of Roche Diagnostics, Mannheim, Germany.
